# Postoperative aspartate aminotransferase to lymphocyte ratio index change is an independent predictor of survival in patients with small hepatocellular carcinoma

**DOI:** 10.1097/MD.0000000000008540

**Published:** 2017-11-10

**Authors:** Chao He, Wei Peng, Chuan Li, Tian-Fu Wen

**Affiliations:** Department of Liver Surgery & Liver Transplantation Center, West China Hospital, Sichuan University, Chengdu, Sichuan, P.R. China.

**Keywords:** aspartate aminotransferase to lymphocyte ratio index, hepatectomy, hepatocellular carcinoma, prognosis

## Abstract

Elevated preoperative aspartate aminotransferase (AST) to lymphocyte ratio index (ALRI) is reported to be a prognostic factor for patients with hepatocellular carcinoma (HCC) after treatment. However, [DELTA] ALRI which represents the change from postoperative ALRI to preoperative ALRI change has received little attention. The present study was designed to evaluate the prognostic value of [DELTA] ALRI in small HCC patients after liver resection.

A retrospective cohort study was performed to analyze 241 patients with small HCC who underwent liver resection. Patients were divided into Group A ([DELTA] ALRI < 0, n = 142) and group B ([DELTA] ALRI ≥ 0, n = 99) according to postoperative ALRI change. Clinical data, overall survival (OS), and recurrence-free survival (RFS) were compared between the 2 groups, and a multivariate analysis was used to identify prognostic factors.

The 1, 3, and 5-year OS rates were 96.5%, 84.9%, and 70.8%, respectively, for group A, and 94.9%, 75.8%, and 59.7%, respectively for group B (*P* = .014). The corresponding 1, 3, and 5-year RFS rates were 78.2%, 54.6%, and 52.3%, respectively, for group A, and 62.6%, 40.1%, 24.5%, respectively, for group B (*P* < .001). The results of univariate and multivariate analysis indicated that [DELTA] ALRI was an independent prognostic factor for both RFS (*P* < .001, hazard ratio [HR] 2.192, 95% confidence interval 1.527–3.147) and OS (*P* < .001, HR 2.381, 95% confidence interval 1.503–3.771).

A positive [DELTA] ALRI after liver resection predicts decreased OS and RFS in patients with small HCC.

## 1 Introduction

Hepatocellular carcinoma (HCC) is one of the most common malignancies and the 3rd leading cause of cancer related death worldwide. Due to a high prevalence of hepatitis B virus (HBV), China alone accounts for 50% of HCC patients in the world, making this disease a great burden on the country.^[1,2]^ The management of liver cancer has improved significantly in the last few decades. Nevertheless, liver resection remains one of the standard treatment methods for small HCC patients who meet the Milan criteria.^[3]^ Despite progress in management in HCC, the incidence of postoperative recurrence for patients with small HCC can be as high as 50% to 70% after liver resection, which is still the main obstacle to satisfactory long-term survival of HCC patients.^[4,5]^

Further efforts to identify the prognostic indicators are necessary. Previous studies have suggested that liver inflammation play a key role in poor liver regeneration,^[6]^ postoperative hepatic failure^[7]^ and the development of HCC,^[8]^ and defective immunity is involved in the development of HCC.^[9]^ Aspartate aminotransferase (AST) is a reliable and sensitive biochemical marker of liver injury. AST to platelet ratio index (APRI) has been developed as a noninvasive index in predicting fibrosis and cirrhosis of liver,^[10]^ and growing evidence demonstrates the relationship between high APRI and poor prognosis of HCC.^[11,12]^ Lymphocyte plays central roles in host’ antitumor immune response, and its presence closely relates to a reduced risk of relapse of HCC. Emerging evidence indicates that absolute lymphocyte count is a strong predictor for recurrence of HCC.^[13,14]^ Recently, aspartate aminotransferase to lymphocyte ratio index (ALRI) was identified as an accurate prognostic indicator for patients with HCC after liver resection and transcatheter arterial chemoembolization.^[15,16]^ However, few studies have elucidated this relationship. [DELTA] ALRI, which represents the change from postoperative to preoperative ALRI, has received little attention. The present study was designed to evaluate the prognostic value of ALRI in patients with HBV related small HCC who underwent liver resection.

## 2 Methods

Between February 2007 and March 2013, 346 patients newly diagnosed with small HCC who met the Milan Criteria underwent liver resection in the Department of Liver Surgery & Liver Transplantation Center of West China Hospital. Diagnosis of all the patients was confirmed by postoperative pathologic report. The degree of liver fibrosis or liver cirrhosis was also determined in postoperative histopathology examination in accordance with the Ishak scoring system. The demographic data, oncological data, hematological tests, liver function tests, HBV markers, and follow-up data were retrieved from our prospectively maintained database. In the present study, our inclusion criteria were as follows: primary HBV related small HCC meeting Milan criteria (solitary tumor <5 cm in diameter or 3 nodules 3 cm in diameter) and no extrahepatic metastasis; adequate reserve liver function (Child–Pugh grade A); appropriate renal function (serum creatinine <124 mmol/L); underwent liver resection as initial treatment. Exclusion criteria included the following: extrahepatic malignancies or massive intrahepatic metastasis; previous resection, TACE, RFA, or liver transplantation; loss to follow up within 3 months after liver resection; poor liver reserve function with a Child–Pugh grade B or C; rupture of HCC; simultaneous splenectomy; clinical symptoms or signs of sepsis or infection at the time of blood sampling for ALRI; poor data integrity. The present study was approved by the Ethics Committee of West China Hospital, Sichuan University.

### 2.1 Definition and calculation of aspartate aminotransferase to lymphocyte ratio index (ALRI)

The routine blood and liver function tests performed within 1 week before liver resection were considered in this analysis. ALRI was calculated with the following formula: AST value (IU/L)/lymphocyte count (10^9/L). The AST upper limit of normal value was defined as 40 IU/L. Postoperative ALRI was obtained at the 1st follow-up visit, 1 month after the operation. Postoperative ALRI change, [DELTA] ALRI was calculated by subtracting the preoperative ALRI from the postoperative ALRI.

### 2.2 Follow-up

All patients were regularly followed up at the 1st, 3rd, and 6th months in the 1st half year after the operation, every 3 months throughout the following 3 years, and every 6 months thereafter. Physical examination, blood cell and differential counts, AFP levels, liver function tests, HBV markers and HBV-DNA levels, and imaging examinations were included when necessary in the follow-up examinations. Antiviral therapy was administered to patients with positive hepatitis B DNA before and after operation. OS time was defined as the interval between the operation and death or the last follow-up. Recurrence-free survival (RFS) time was defined as the time interval between the operation and the 1st incidence of detectable recurrence. The last follow-up date was the end of Nov 2016.

### 2.3 Statistical analysis

Statistical analysis was conducted with SPSS software, version 21.0 (SPSS Company, Chicago, IL). Categorical data were compared by the chi-square test or Fisher exact test. Continuous variables were compared by independent *t* test for normal distributed data or Mann–Whiney *U* test for abnormal distributed data. The OS and RFS were analyzed by the Kaplan–Meier method, and the differences were analyzed by a log-rank test. Variables with significance on univariate analysis were further evaluated by multivariate Cox proportional hazards regression analysis. All *P* values were 2 sided, and a significant difference was considered when the *P* value was <0.05.

## 3 Results

**Figure 1:**
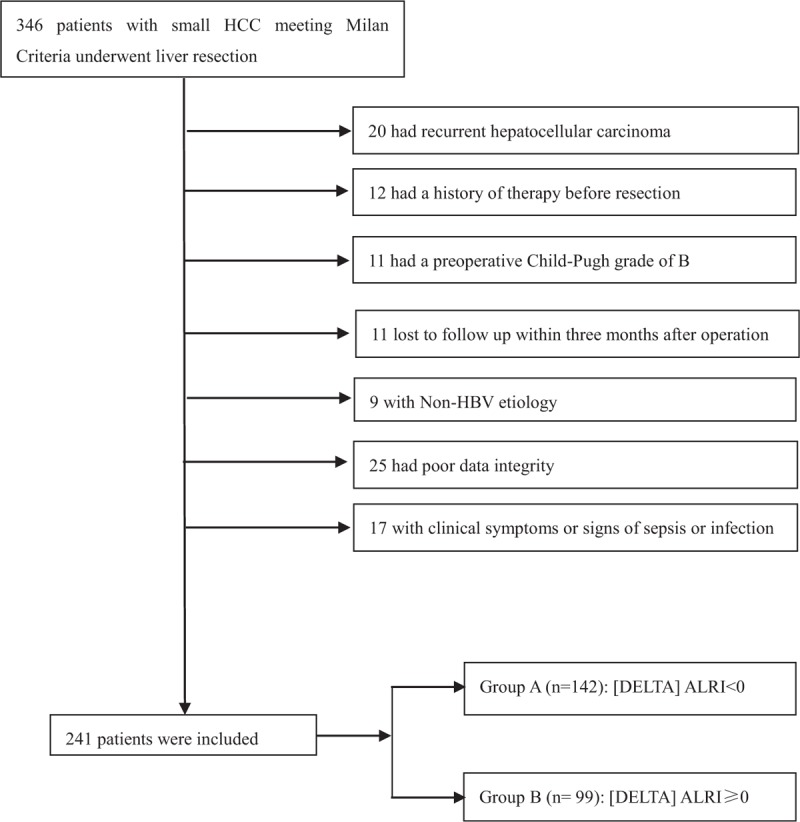
Flowchart of the process for patients’ selection.

Based on the inclusion and exclusion criteria, a total of 105 patients were excluded from the present study. Finally, a retrospective analysis was made in 241 patients who received liver resection as initial treatment during February 2007 and 2013 in our center. Details about patient selection are shown in Fig. 1. Among them, there were 210 men and 31 women (12.8%), with the mean age of 50.3 years (range, 21–78 years). A total of 108 patients (44.8%) had a nodule lesser than or equal to 3 cm in diameter, and 133 patients (55.2%) had a nodule 3 to 5 cm in diameter. Of the total, 207 patients (85.9%) had only 1 nodule, whereas 34 patients (16.4%) had 2 or 3 nodules. During the follow-up period, 151 patients (62.7%) recurred and 88 patients (36.5%) were dead. The mean value of preoperative ALRI was 32; there were 169 patients (70.1%) with preoperative ALRI less than 32, and 72 patients (29.9%) with preoperative ALRI 32 or more. One month after operation, the ALRI was decreased in 142 patients (58.9%) and increased in 99 patients (41.1%) comparing with the preoperative ALRI. The characteristics of the decreased group and increased group were described in Table 1. Patients in group A tend to have single nodule. The mean preoperative ALRI value was lower and the mean postoperative ALRI value was higher in group B (*P* < .001 for both). No other association was observed.

**Table 1:**
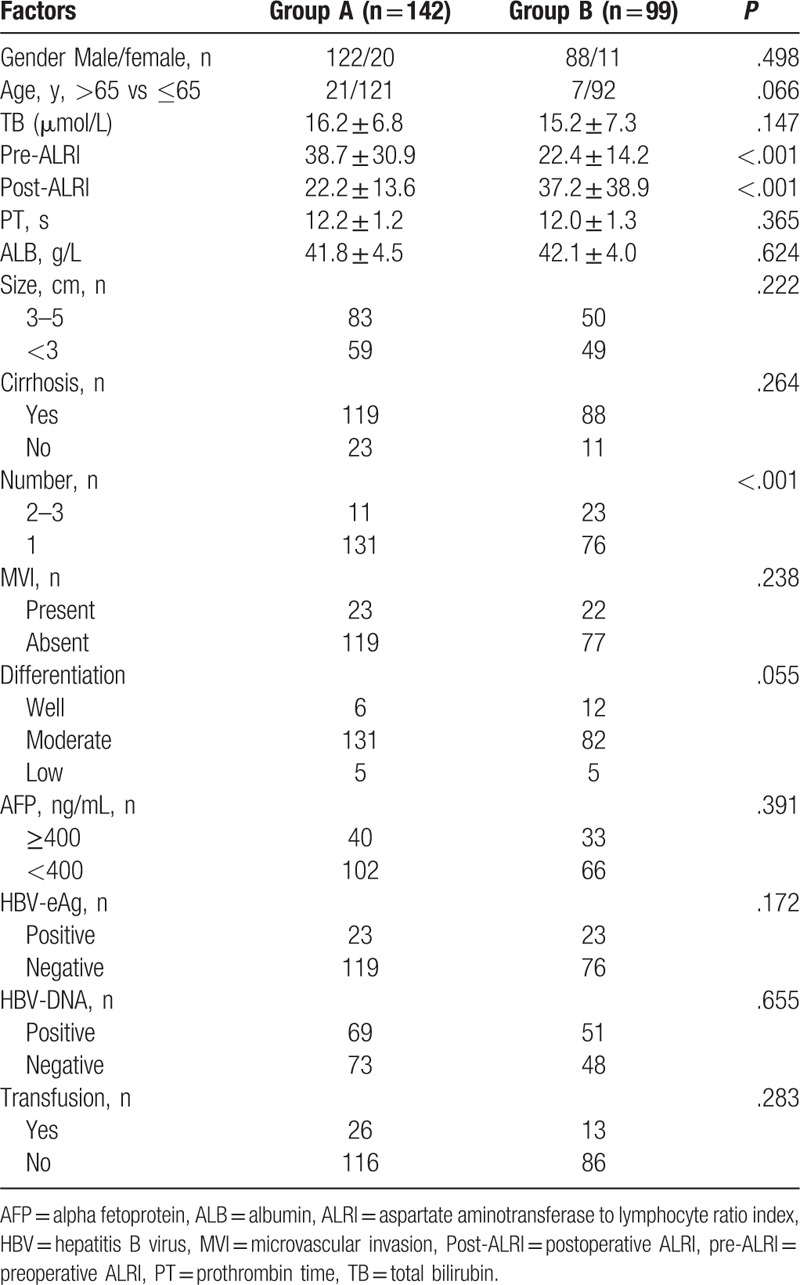
Baseline characteristics of patients according to postoperative ALRI change.

### 3.1 Impact of ALRI on OS

**Figure 2:**
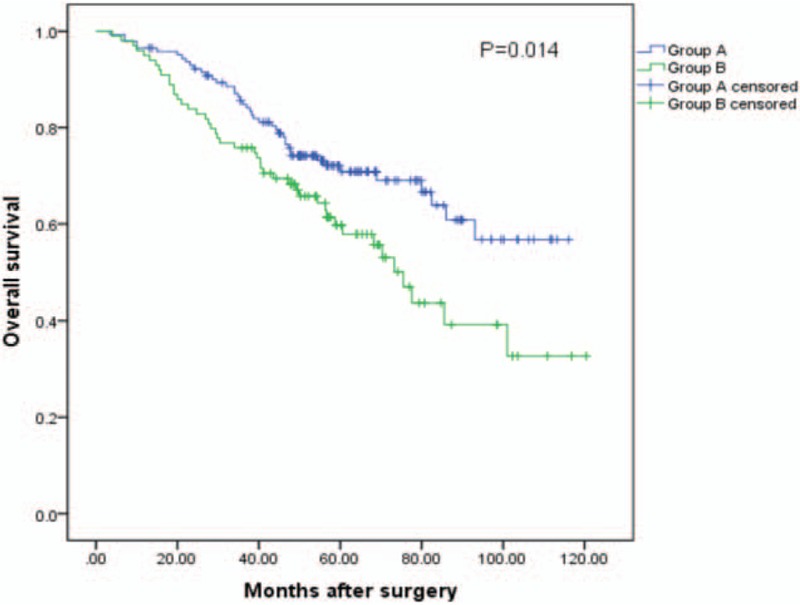
Relationship between [DELTA] ALRI and overall survival (OS) of small HCC patients after liver resection. The difference of OS between 2 groups was significant (log-rank test, *P* = .014). [DELTA] ALRI = postoperative ALRI change, HCC = hepatocellular carcinoma.

The median follow-up time was 54.2 months (range, 3.4–120.4). During the follow-up, a total of 88 patients died. The 1-, 3- and 5-year OS rates of the 241 patients were 95.9%, 81.1%, and 66.3%, respectively. The 1, 3, and 5-year rates OS were 96.5%, 84.9%, and 70.8%, respectively, for group A and 94.9%, 75.8%, and 59.7%, respectively, for group B (log-rank test, *P* = .014). (See Fig. 2).

### 3.2 Impact of ALRI on RFS

**Figure 3:**
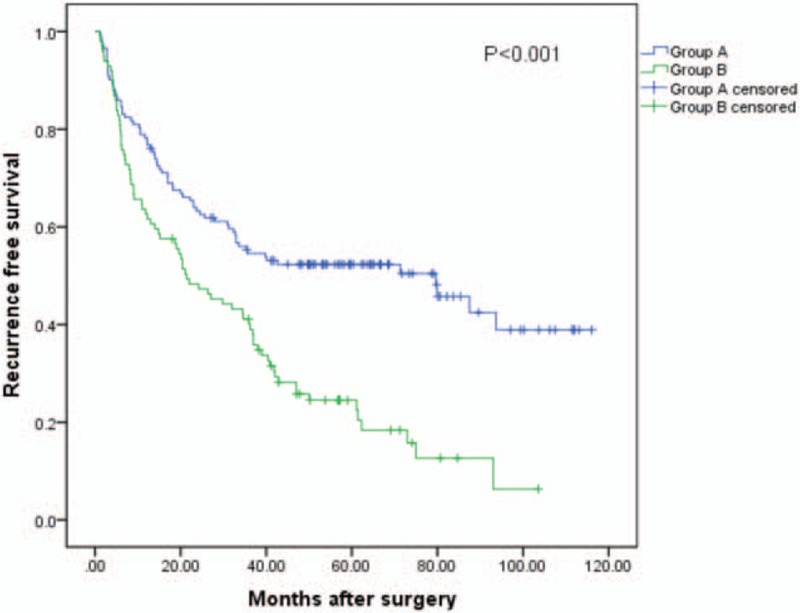
Relationship between [DELTA] ALRI and recurrence free survival (RFS) of small HCC patients after liver resection. The difference of RFS between 2 groups was significant (log-rank test, *P* < .001). [DELTA] ALRI = postoperative ALRI change, HCC = hepatocellular carcinoma.

The cumulative 1, 3, and 5-year RFS rates among all the patients were 71.8%, 48.6%, and 41.0%, respectively. The 1, 3, and 5-year RFS rates were 78.2%, 54.6%, and 52.3%, respectively, for group A, and 62.6%, 40.1%, and 24.5%, respectively, for group B (log-rank test, *P* < .001). (See Fig. 3).

### 3.3 Independent predictors for OS

The results of the univariate and multivariate analyses of the OS were illustrated in Table 2. The univariate analysis demonstrated that cirrhosis (*P* = .090), tumor number (*P* = .078), microvascular invasion (MVI) (*P* = .003), AFP (*P* = .065), transfusion (*P* < .001), preoperative AST (*P* < .001), postoperative AST (*P* = .02), preoperative lymphocyte (*P* = .041), postoperative lymphocyte (*P* = .001), preoperative ALRI (*P* < .001), and [DELTA] ALRI (*P* = .016) were significant predictors for OS. On the multivariate analysis of OS, microvascular invasion (*P* < .001, hazard ratio [HR] 2.570, 95% confidence interval [95% CI] 1.604–4.117), AFP (*P* = .031, HR 1.621, 95% CI 1.045–2.515), transfusion (*P* = .017, HR 1.900, 95% CI 1.124–3.213), preoperative ALRI (*P* < .001, HR 1.016, 95% CI 1.009–1.023), [DELTA] ALRI (*P* < .001, HR 2.381, 95% CI 1.503–3.771) remained significant independent predictors of OS.

**Table 2:**
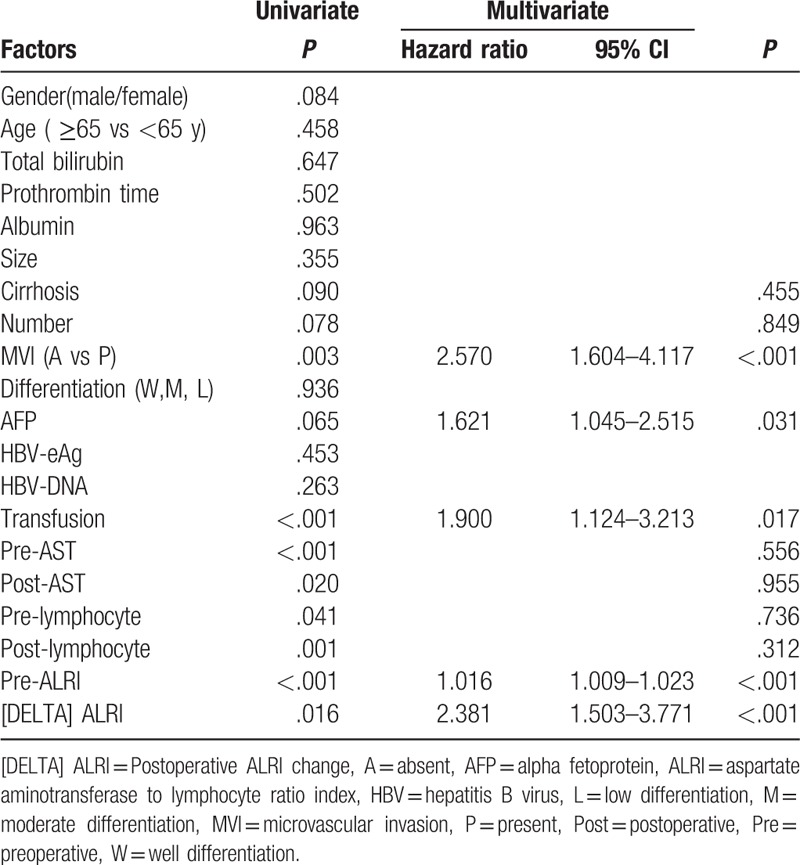
Univariate and multivariate analysis of prognostic factors of overall survival.

### 3.4 Independent predictors for RFS

The results of the univariate and multivariate analyses of the RFS were illustrated in Table 3. The univariate analysis suggested that albumin (*P* = .099), cirrhosis (*P* = .016), tumor number (*P* = .001), MVI (*P* = .047), HBV-eAg (*P* = .015), Hepatitis B virus DNA (*P* = .009), transfusion (*P* = .044), preoperative AST (*P* = .007), postoperative AST (*P* = .022), postoperative lymphocyte (*P* = .033), preoperative ALRI (*P* = .026), [DELTA] ALRI (*P* = .022) were significantly associated with RFS. The multivariate analysis showed that tumor number (*P* = .006, HR 1.814, 95% CI 1.190–2.765), MVI (*P* < .001, HR 2.219, 95% CI 1.500–3.283), HBV-eAg (*P* = .023, HR 1.553, 95% CI 1.063–2.269), preoperative ALRI (*P* < .001, HR 1.012, 95% CI 1.007–1.018), and [DELTA] ALRI (*P* < .001, HR 2.192, 95% CI 1.527–3.147) were independently associated with RFS of small HCC patients after liver resection.

**Table 3:**
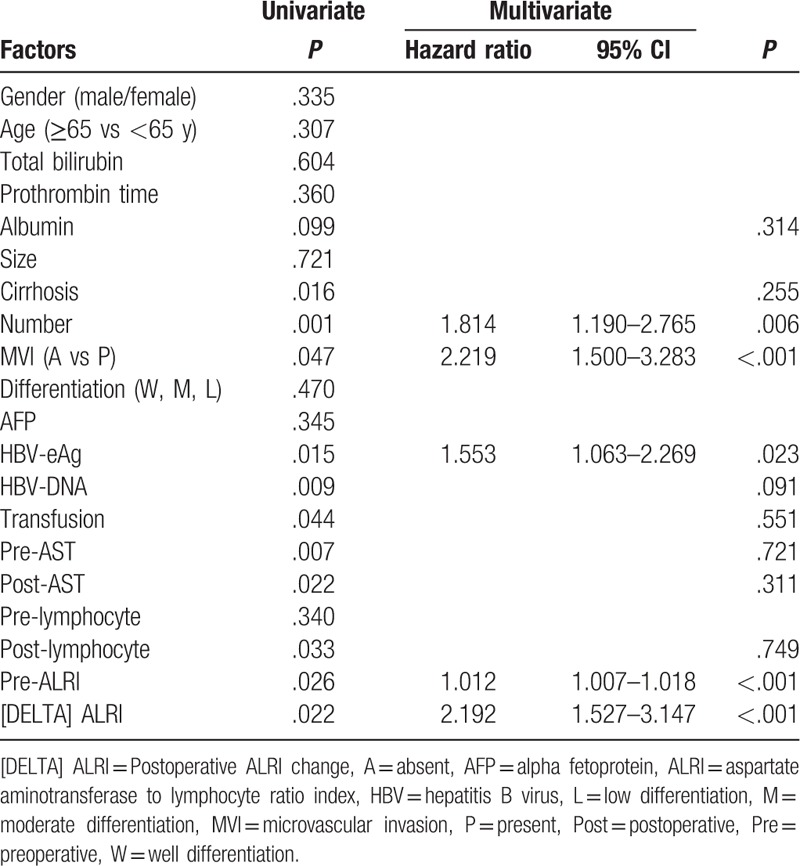
Univariate and multivariate analysis of prognostic factors of recurrence-free survival.

## 4 Discussion

HCC is a great burden for human health worldwide. Searching of valid indicators to predict HCC prognosis is of great importance in therapeutic options of HCC. Convenient, easily-obtained, low cost, reliable and noninvasive biochemical markers for HCC are needed. Over the decades, the prognostic significance of several index including neutrophil-to-lymphocyte ratio (NLR), prognostic nutritional index (PNI), and aspartate aminotransferase-to-platelet ratio index (APRI), and percentage of lymphocyte in post-surgery patients with HCC has been validated.^[11,17,18]^ However, few study focused on the value of ALRI in predicting prognosis of HCC. Junfei Jin et al^[15]^ first reported that ALRI can serve as a predictor of survival in patients with HCC after liver resection, and preoperative ALRI >25.2 predicted poor OS and disease free survival. Similarly, preoperative ALRI >57 was demonstrated to be an independent risk factor for poor OS in patients with HCC after TACE.^[16]^ However, these studies focused on pretreatment ALRI, whereas the significance of the differences between preoperative and postoperative ALRI has not been examined.

The results of the present study suggest that preoperative ALRI, MVI, [DELTA] ALRI are independent risk factors of both OS and RFS. These findings indicated that [DELTA] ALRI might represent a reliable and stable prognostic factor in patients with small HCC and might have valuable applications in clinical practice for determining postoperative treatment in patients with small HCC.

The results of our study were consistent with previous findings. We found that patients with a low preoperative ALRI had a more favorable prognosis after liver resection. However, the hazard ratio of preoperative ALRI is relatively low. We considered that a universal and accurate cutoff value of preoperative ALRI which meets the needs of all medical centers was not possible, because different patient populations from different medical centers inevitably use different cut-off values. In the present study, for the first time, we studied [DETLTA] ALRI, which would not be affected by the ALRI cutoff value and might reflect the dynamic change of the host inflammatory response and the immune response from preoperative to postoperative periods. Ultimately, we determined that [DELTA] ALRI was an independent prognosis indicator for patients with small HCC after liver resection and might be better than preoperative ALRI in predicting prognosis of small HCC.

The significance of ALRI in predicting prognosis of patients with HCC after liver resection is consistent with the pathogenesis of HCC. ALRI was calculated using AST and lymphocyte levels. AST is a reliable and sensitive biochemical marker of liver injury. Advanced diseases involving mitochondrial injury of hepatocytes, may lead the release of AST to serum.^[19]^ And also fibrosis and cirrhosis can reduce the sinusoidal clearance of AST.^[20]^ Further, a higher AST level was showed to correlate with a greater influx of hepatitis B virus, which associates with decreased overall survival in HCC patients.^[21]^ Therefore, the AST level indicates the degree of liver damage. Conversely, multiple studies have reported that lymphocytes play a key role in antitumor immune responses.^[22,23]^ Previous investigations indicated that CD8 (+) cytotoxic T-cells play a central role in eliminating tumors and that regulatory T cells may suppress the immune reaction. The functional impairment of CD8 (+) T-cells and CD4 (+) cytotoxic T-cells was also associated with poor survival rates and high recurrence of HCC after liver resection.^[23]^ Further, decreased absolute lymphocyte level in patients are associated with high recurrence incidence and poor OS.^[13,14,24]^ Several factors may account for lymphopenia in HCC patients. As the majority of HCC patients are with liver fibrosis or cirrhosis.^[25]^ Depletion of naive and memory lymphocyte, defective thymopoiesis due to accelerated aging and atrophy of the thymus, impaired peripheral compensatory proliferation and spleen sequestration, cell consumption related to activation-driven bacterial translocation, and increased apoptosis all are possible etiological factors.^[26,27]^

Theoretically, ALRI is expected to decrease after liver resection, because patients who were HBV-DNA positive were prescribed with anti-viral therapy, which might ameliorate liver inflammation and improve liver functional reserves in patients with chronic hepatitis.^[28]^ Furthermore, impaired immunity has been reported in HCC patients.^[29,30]^ Removal of HCC theoretically alleviates immune suppression in HCC patients. Therefore, HCC patients with decreased ALRI after liver resection were more likely to have better liver function and immune status than HCC patients with increased ALRI, and hence could have better prognosis.

When comparing the demographic data of the decreased and increased [DELTA] ALRI groups, we found that the mean preoperative ALRI value was lower and the mean postoperative ALRI value was higher in the [DELTA] ALRI increased group. This result suggested that inflammatory and immune response could change after removal of the tumor, and that this dynamic change may result in a different prognosis. We also noted that decreased [DELTA] ALRI correlates significantly with the presence of tumor number, which suggested that an increase in postoperative ALRI may correlate with multi-nodular type of HCC.

We acknowledge the limitations of our study. First, the present study was a retrospective cohort study conducted at a single medical center, and the study population was comprised primarily of HBV-related small HCC cases that only have been internally validated. Second, some clinic-pathological characteristics, including capsule state, portal hypertension, and TNM stage, were not available in our report. Third, other inflammation markers, such as alanine transaminase (ALT), C reactive protein, and interleukin were not evaluated in our study as well as immunity indicators, such as CD4+ and CD8+ subset. Therefore, further prospective, well-designed studies with larger samples are needed to verify the prognostic value of [DELTA] APRI in HCC, and the potential mechanism underlying this association.

In conclusion, we developed a noninvasive, low cost, easily assessable and reproducible prognostic parameter, [DELTA] ALRI. The results of the present study suggest that positive [DELTA] ALRI is an independent predictor of decreased OS and RFS after liver resection. HCC patients with positive [DELTA] ALRI should, therefore, be closely monitored and timely postoperative therapeutic intervention should be conducted to improve their long-term survival.
